# Heat-Related Illness and Heatstroke: A Narrative and Clinical Review for Emergency Clinicians

**DOI:** 10.7759/cureus.107294

**Published:** 2026-04-18

**Authors:** Rachel E Bridwell, Brit Long, Alex Koyfman, Aaron J Lacy

**Affiliations:** 1 Emergency Medicine, Uniformed Services University of the Health Sciences, Bethesda, USA; 2 Emergency Medicine, University of Virginia, Charlottesville, USA; 3 Emergency Medicine, University of Texas Southwestern Medical Center, Dallas, USA; 4 Emergency Medicine, Washington University School of Medicine, St. Louis, USA

**Keywords:** classical, cooling, exertional, heat illness, heatstroke

## Abstract

Heat-related illness is a spectrum of conditions that can have a high rate of morbidity and mortality. This review highlights the pearls and pitfalls of heat-related illness, particularly heatstroke, including presentation, diagnosis, and management in the emergency department based on current evidence. Heatstroke represents the most severe form of heat-related illness and is characterized by core temperature above 40°C with central nervous system dysfunction. Both classic and exertional forms share similar pathophysiology driven by thermoregulatory failure, systemic inflammation, and multiorgan injury. Emergency department management centers on rapid recognition and immediate cooling, as outcomes correlate with duration and magnitude of hyperthermia. Cold-water immersion remains the gold standard for rapid cooling; however, evaporative and conductive methods such as ice sheets, tepid water misting with fanning, and administration of chilled IV fluids are effective alternatives when immersion is impractical. Continuous core temperature monitoring is essential to avoid overcooling, with a target of 39°C. Sedation should be achieved chemically rather than through physical restraint to prevent further heat generation, and vasopressors should be avoided unless hypotension persists despite aggressive cooling and volume resuscitation. Diagnostic testing should not delay cooling, though evaluation for multiorgan dysfunction and mimics such as sepsis or toxicologic causes is critical. In conclusion, an understanding of heat-related illness and heatstroke can assist emergency clinicians in diagnosing and managing this potentially deadly disease.

## Introduction and background

Heat-related illness is a spectrum of diseases that range from self-limited conditions, including heat rash, heat edema, heat syncope, heat cramps, and heat exhaustion, to severe conditions, including heat stroke, which is associated with severe morbidity and mortality (Table 1) [1-4]. An imbalance between heat clearance and heat generation results in heat-related illness. This imbalance may cause only mild symptoms, in which normal human physiology can compensate, or may progress to a state of profound dysregulation in which the human body can no longer compensate, termed "heat stroke." Heat stroke is defined by a temperature greater than 40°C, neurologic abnormalities (e.g., change in mental status), and the result of exposure or exertion [1-4]. Heat stroke is typically categorized as classic, also termed non-exertional or passive, and exertional. Classic heatstroke often occurs as an epidemic among elderly and pediatric patients due to physiological, medical, and social factors. Exertional heat stroke is associated with strenuous activity and most commonly involves athletes, laborers, and soldiers [1-4]. Table 1 summarizes heat-related illness, including classic and exertional heat stroke. Given significant morbidity and mortality, as well as a rising incidence, the emergency physician must be prepared to care for the patient with heat illness. This narrative review will cover heat-related illness, with a specific focus on heatstroke, given its significant morbidity and mortality. 

**Table 1:** The spectrum and characteristics of heat-related illnesses [1-4]

Heat-Related Illness	Characteristics
Heat rash (miliaria rubra, prickly heat)	Pruritic, maculopapular, erythematous rash most commonly found under clothed areas; due to occlusion and inflammation of sweat ducts
Heat edema	Lower extremity dependent edema; typically benign, self-limited; result of increased hydrostatic pressure, cutaneous vasodilation; vascular leak
Heat cramps	Spasmodic, involuntary skeletal contractions due to a relative muscle electrolyte deficiency; rare complications include rhabdomyolysis; usually occurs during or after exercise as the muscles cool
Heat syncope	Brief syncopal episode with rapid return to baseline; orthostatic syncope due to cutaneous vasodilation resulting in blood pooling; can be due to inadequate liquid or electrolyte intake
Heat exhaustion	Normal or elevated core temperature but less than 40 degrees Celsius; normal mental status but symptoms/signs include weakness, syncope, nausea and vomiting, tachycardia; may progress to heat stroke if not treated or be complicated by rhabdomyolysis, renal injury, disseminated intravascular coagulation
Classic heat stroke	Temperature over 40° Celsius, CNS dysfunction; due to excessive heat absorption and poor heat dissipation; poor physiologic mechanism for heat regulation (elderly, pediatrics, medications that impair physiologic response; psychiatric/socioeconomic barriers; usually occurs in epidemic in sedentary or chronically ill patients; sweating may be absent; rhabdomyolysis and renal failure are unusual; liver dysfunction mild; CK mildly elevated; calcium and phosphorus typically normal
Exertional heat stroke	Temperature over 40° Celsius,increasesfunction; due to excessive generation that overwhelms heat dissipation; typically occurs in athletes, laborers, or soldiers; hotter environment, wearing gear, poor conditioning, prior history of heat illness, lack of heat acclimatization increase risk; sweating typically present; rhabdomyolysis and renal failure are more common; liver dysfunction severe; CK markedly elevated; calcium low and phosphorus high

Methodology

This review was conducted in a narrative format, with a focus on specific questions and clinical scenarios relevant to the emergency clinician. The author group discussed specific areas of need relating to heat illness management for the emergency clinician and ultimately, through consensus, decided on the topics included in the review. Group members subsequently performed independent literature reviews for the different sections of the review and used them to form the content of the manuscript. Searches were restricted to studies published in English, with a focus on emergency medicine, prehospital, and critical care literature. Studies from the last decade were of particular interest, with searches and manuscript development completed in June of 2025. This narrative review synthesizes consensus guidelines and observational evidence, and no formal meta-analysis was performed due to heterogeneity in study designs and settings. In total, 74 sources were used to inform the content of the manuscript, including sources added after peer review.

Pathophysiology/Epidemiology

Pathophysiology 

The body controls temperature through several mechanisms. The central nervous system (CNS), including the hypothalamus and peripheral receptors in the skin, communicates to control heat loss [1,2,5-7]. A temperature gradient between the skin and the body core allows for heat dissipation when the core temperature is greater than the surface temperature, with reduced dissipation with a narrower gradient. Once the external environment reaches 35°C, known as the critical temperature, the gradient sufficiently narrows and heat loss is significantly impacted [3]. There are several mechanisms by which the body absorbs and loses heat. Conduction is the transfer of heat by direct contact between two different surfaces [8]. Convection is the transfer of heat from a solid or liquid to ambient air or moving liquid [8]. Evaporation is the loss of heat as liquid water changes to gas. Air movement over the skin is typically necessary for evaporation to occur [8]. Finally, radiation is the transfer of heat by electromagnetic waves [8]. As the ambient temperature and humidity increase, convection and evaporation become less effective in dissipating heat. As the core temperature begins to elevate, the hypothalamus signals blood vessels to vasodilate, shunting blood to the skin to assist in removing heat [8]. For each 1°C increase in blood temperature, the hypothalamus can cause a resultant increase in vascular flow to the skin by up to 8 L per minute [9,10]. Cardiac output also increases to improve blood flow to the skin and metabolism in the muscles. The resultant cutaneous cardiovascular shunting reduces renal and gastrointestinal blood flow by upwards of 30% [11]. The hypothalamus also increases sweat production through the eccrine and apocrine sweat glands, though this can be reduced by prescription and illicit drugs [12]. Finally, the hypothalamus provides behavioral responses to reduce exertion, remove clothing, and seek a sheltered area [12]. However, as the temperature continues to rise and the critical temperature threshold is reached, the ability to dissipate heat is compromised. Thermal production then outpaces the ability to dissipate heat, resulting in heat illness. The acute phase results in a systemic inflammatory response with the release of cytokines, interleukins, and proteins that increase mucosal permeability [13]. This, in turn, results in the release of endotoxins from the GI tract, further injuring tissues and endothelium [13]. As the temperature continues to increase in the body, proteins begin to denature, resulting in cell apoptosis and temperature-induced coagulopathy with severe end-organ dysfunction [14]. Direct heat injury may also occur, resulting in necrosis [14]. Ultimately, this constellation of events leads to multi-organ failure, most notably acute renal failure, GI ischemia, myocardial injury, and CNS dysfunction. This culminates in severe hemodynamic compromise, cerebral injury, and death [15].

Epidemiology

Heat-related illness is increasing in incidence due to climate change and patient factors, including growing rates of obesity [16]. The incidence of heat stroke in the U.S. includes over 1.3 ED visits per 100,000 population, with the highest rates in the south [17]. The incidence increases with age and is highest in those over age 80 years at 4.45 visits per 100,000 [17]. Other than the elderly, other populations at significant risk include those with medical comorbidities, pediatric patients, those with limited access to cooling, psychiatric illness, and occupational or recreational exposure [17]. There are a variety of risk factors, which can be divided into non-modifiable and modifiable factors. While the most common heat-related illness that presents to the ED is heat exhaustion, heat stroke is the most dangerous, with mortality rates ranging between 10-63% [18-23]. 

## Review

Presentation and emergency department evaluation

Presentation

Hyperthermia in isolation is a physiological process in response to elevated ambient temperatures or internally generated heat due to strenuous physical exercise and is only pathological in combination with symptoms. The associated spectrum of heat illnesses that result from hyperthermia ranges from mild to severe, with severe presentations occurring with a core temperature greater than 40°C [2].

Mild heat illness encompasses heat rash, heat edema, and heat cramps. Heat rash, also known as prickly heat or miliaria rubra, presents as an erythematous papular, papulovesicular, or pustular eruption of the epidermis or dermis on an erythematous background with associated pruritus and stinging [24]. These eruptions typically occur in areas of contact with clothing (e.g., neck, trunk) and in the flexural surfaces of the body [24]. Heat edema presents with dependent extremity swelling and facial flushing and is a self-limiting condition. Heat cramps are involuntary, painful muscle contractions of large muscle groups [24].

Moderate heat illness involves heat syncope and heat exhaustion. Heat syncope presents as a transient loss of consciousness with rapid return to baseline in the setting of heat exposure [1]. When syncope occurs immediately after cessation of exercise, it is termed exercise-associated collapse and is due to lower-extremity venous pooling, peripheral vasodilation, volume depletion, and decreased vasomotor tone [5]. Heat exhaustion refers to a spectrum of symptoms that include fatigue, malaise, intense thirst, weakness, discomfort, anxiety, dizziness, and gastrointestinal symptoms, often a precursor to heat stroke [1].

Heat stroke is the most severe manifestation of heat illness and a medical emergency, classified into classical and exertional types. For both pathologies, heat stroke presents as a core temperature above 40°C with evidence of CNS dysfunction [2]. Neurologic symptoms indicating heat stroke include altered mental status and loss of consciousness. Patients present with altered mental status, seizures, coma, tachycardia, hypotension, and hyperventilation [1].

ED Evaluation

ED evaluation is a dichotomous process that centers around both assessing the current temperature and hemodynamic state of the patient, as well as identifying any potential etiologies outside of exertional heat stroke. An initial set of vital signs is crucial, which, in addition to hyperthermia, may show tachycardia, hypotension, and tachypnea. While obtaining an esophageal temperature is the gold standard, a rectal temperature is far more expedient in the ED. Once life-threatening hemodynamics and cooling are addressed, a temperature-sensing Foley or esophageal probe is ideal for continuous core temperature monitoring and to avoid overshooting on cooling therapies [25]. A point-of-care glucose should be acquired with initial vitals to rule out hypoglycemia as a concomitant reason for alteration in mental status. Removing all clothes and layers helps with passive cooling and allows for a thorough physical exam [26]. As outlined above, heat stroke patients may present with a variety of neurological findings, ranging from altered mental status, seizures, and coma. Focal neurologic deficits are more likely to be seen with intracranial catastrophe contemporaneous with heat stroke or hemorrhagic hypothalamic lesions [27-29]. ED clinicians should perform thorough posterior and integumentary exams, looking for any evidence of trauma, skin lesions, stigmata of toxicology, cellulitis, or necrotizing soft tissue infections.

While laboratory evaluation should not delay cooling, a comprehensive metabolic panel with calcium, magnesium, and phosphate is key. An independent risk factor for death is hypernatremia exceeding 145 mEq/L. Other derangements, such as hyponatremia, inversely altered phosphate, and high or low potassium, magnesium, and calcium, portend a higher risk of mortality [30-32]. Liver-associated enzymes should be acquired, as the risk of mortality is associated with an aspartate aminotransferase (AST) level greater than 1000 units [33]. Creatinine kinase and elevated troponin may be noted in heat stroke patients, though they may represent concomitant rhabdomyolysis versus skeletal muscular breakdown due to hyperpyrexia, as opposed to true cardiac or renal dysfunction [34]. In a study comparing heat stroke patients who died versus those who survived, the fatal heat stroke group was noted to have higher lactate, troponin, and creatinine, though this group had a delay in cooling [35]. Coagulation studies, including fibrinogen, may be useful, as heat stroke can produce disseminated intravascular coagulopathy [36]. The many lab derangements produced in the setting of heat stroke highlight the global shock state that can result [35]. An electrocardiogram should also be acquired to assess for any dysrhythmia, especially for patients with suspected toxicologic etiologies (e.g., sodium channel blockers or sympathomimetics). Similarly, a urine drug screen may help identify any toxicologic ingestions, although these can be prone to false positives and negatives. If the etiology is uncertain, a thyroid-stimulating hormone may help identify thyrotoxicosis or thyroid storm as a possible etiology [37]. Though some patients may undergo advanced imaging for intracranial catastrophe concerns, routine head computed tomography or magnetic resonance imaging is not recommended in initial evaluation, as they may interfere with the primary mission of rapid cooling.

Emergency department management

Overview of ED Management

The ED management of heat stroke focuses on cooling the patient rapidly while terminating any causative etiologies [38,39]. Cooling therapy starts in the prehospital environment with removal from the hot location and initiation of passive cooling. This can be accomplished with any means available, including pre-staged ice sheets, tarp-assisted cooling, immersion tanks, and even helicopter downdraft [40]. Other passive cooling options include oral administration of cooled fluids, immersion in ice water baths, spraying lukewarm water with a fan blowing, cooling blankets, external pads with adaptive water-based cooling systems (e.g., Arctic Sun), and chilled air via the respiratory tract through a high-flow nasal cannula +/- endotracheal tube [1,41]. A cold-water submersion (also termed "cold water immersion") bath is considered the ideal cooling method with the fastest cooling rates, recommended by the Wilderness Medical Society with a grade 1A recommendation [1]. However, this method may be limited by equipment as well as invasive monitoring required for hemodynamically unstable patients [1]. Developing cold-water immersion protocols can help with the implementation and care of the critically ill patient suffering from heat stroke [39]. Unlike warmed fluids for hypothermia, cooled fluids for hyperthermia are more effective due to a larger temperature gradient between fluids and core temperature, decreasing the core body temperature by 1°C per cooled liter or approximately a 2°C decrease with a 30-40 cc/kg bolus [42,43]. Active cooling measures include intracavitary lavage, vascular cooling catheter placement, and extracorporeal membrane oxygenation, which are more likely to occur in obtunded and intubated patients at the extremes of hyperthermia. Active cooling has demonstrated increased rates of survival as compared to passive cooling [38,44].

While simultaneously cooling the patient, controlling agitation is equally paramount, as it increases the risk of rhabdomyolysis and thermogenesis, hindering vital treatments. Clinicians should avoid physical restraints, which would only further harm the patient, and instead use chemical sedation with benzodiazepines and potentially ketamine for quick control. Avoidance of fentanyl is crucial for patients in whom serotonin syndrome is suspected [38,44]. Additionally, termination of any shivering reflex is key to preventing any additional thermogenesis. While shivering in intubated patients will be controlled with a long-acting paralytic in the acute phase, propofol and dexmedetomidine are the preferred sedation agents over benzodiazepines for continual blunting of the shivering reflex. Analgesic doses of ketamine and magnesium can also be used as needed to mitigate the shivering reflex [38,45,46].

Rapid cooling helps to decrease the core body temperature to 39°C as fast as possible. Rapid cooling is essential, as the duration and degree of hyperthermia are directly linked to CNS injury and survival [38,39]. Stopping cooling at 39°C prevents overshooting (leading to shivering), and continuous monitoring should occur even after 39°C is reached to follow the patient's clinical course closely.

Patients with heat stroke often require concurrent airway management. Considerations for intubation include refractory agitation, concomitant respiratory failure, acquisition of diagnostics (e.g., MRI, lumbar puncture), interference of respiratory mechanics secondary to rigidity, and facilitation of termination of status epilepticus [45]. During advanced airway management, ED clinicians should avoid succinylcholine to avoid worsening hyperthermia or adding a new confounding issue of rare, but possible, malignant hyperthermia [47]. Given the risk of multiorgan dysfunction and high associated morbidity and mortality, patients with heatstroke should be admitted to an intensive care unit.

Specific considerations, pearls, and pitfalls for management of heat-related illness

What Are the Classifications of Heatstroke?

Heat stroke is most commonly defined as a core body temperature above 40°C, in conjunction with hot, dry skin and central nervous system abnormalities including delirium, convulsions, and coma [48]. Though there is no universally recognized definition of heat stroke, variations in temperature criteria are presented in the literature. Other commonly referenced sources cite temperature criteria of core body temperature >40.6°C, any body temperature >40°C, documented evidence of cooling before temperature recording, and any history of environmental heat exposure [35].

Heat stroke is divided into two classifications based on the underlying pathophysiology: classical heat stroke and exertional heat stroke. Classical heat stroke occurs from passive exposure to environmental heat in patient populations with compromised behavioral and physiological compensatory mechanisms to heat exposure [1]. Some prefer to use the term ‘non-exertional’ or ‘passive’ to help better characterize the distinction from exertional heat stroke. Exertional heat stroke occurs secondary to internal heat generated by strenuous physical exertion, with or without exposure to elevated ambient environmental temperatures [1]. *What Are Concomitant Etiologies or Heat Stroke Mimics That Can Cloud the Diagnostic Picture?*

The differential diagnosis for hyperthermia with central nervous system derangement is broad and can be found in Table 2. Any pathology that causes changes in mentation associated with elevated temperature can be mistaken for heat stroke, most notably a plethora of toxicologic emergencies. Other major categories include neurologic emergencies, endocrine pathology, infection, and hematologic derangements. 

**Table 2:** Differential diagnosis for heat stroke [1,4,9,13,37]

Category	Considerations for differential
Toxicologic	Alcohol withdrawal, sympathomimetic toxicity, anticholinergic toxicity, salicylate toxicity, malignant hyperthermia, neuroleptic malignant syndrome, serotonin syndrome
Neurologic	Central nervous system infection, hypothalamic stroke (ischemic
Endocrine	Thyrotoxicosis, pheochromocytoma
Infectious	Central nervous system infection, sepsis
Hematologic	Deep venous thrombosis, pulmonary embolism

What Are the Primary Risk Factors for Heatstroke? 

Overall risk factors for heatstroke can be broken down into non-modifiable and modifiable factors. Non-modifiable risk factors include extremes of age, anhidrosis, skin disease, spinal injury, endocrine disorders, neurological disorders, and hereditary disorders (e.g., sickle cell disease). Modifiable risk factors include dehydration, medication or drug use, infection, obesity, exposure to hot and/or humid temperatures, and prolonged exertion. When comparing classic and exertional heatstroke, there are disparate but important risk factors for both, which help identify this disease earlier in the ED course, which can be found in Table 3. Due to reduced thermoregulatory capacity of underlying medical conditions or lack of support systems, classic heatstroke is predisposed to affect the elderly and prepubertal children in times of elevated ambient temperatures. The elderly are particularly susceptible due to underlying conditions and medication side effects. Exertional heat stroke typically affects younger adults when strenuous physical exertion is combined with elevated ambient temperatures, such as soldiers or day laborers.

**Table 3:** Primary risk factors for classical and exertional heat illness [1-8]

Component	Classic Heatstroke	Exertional Heatstroke
Age	Elderly, prepubertal children	Young
Inciting Event	Elevated ambient temperature	Strenuous physical exertion, +/- elevated ambient temperature
Demographic	Isolated, inadequate support system	Athletes, first responders, agricultural workers, military personnel
Environmental	Lack of climate control systems	Endurance races, military training events
Physiologic	Reduced thermoregulatory capacity due to underlying medical conditions	Poor cardiovascular fitness, lack of acclimatization to ambient temperature, dehydration
Medical Conditions	Cardiovascular disease, diabetes mellitus, congenital disorders (ectodermal dysplasia, idiopathic anhidrosis)	Obesity, viral/bacterial infection, dehydration, sleep deprivation, sweat-gland dysfunction, previous heat injury, sickle cell trait
Medications/Substances	Beta blockers, calcium channel blockers, anticholinergics, antihistamines, salicylates, thyroid agonists, laxatives, sympathomimetic medications, tricyclic antidepressants, selective serotonin reuptake inhibitors.	Alcohol abuse, amphetamine abuse, MDMA, cocaine, PCP, LSD, synthetic cathinones, nutritional supplement use

What Testing Is Recommended for Heatstroke?

The diagnosis of heatstroke is made based on 1) a core temperature measurement greater than 40°C and 2) clinical manifestations of central nervous system derangement [2]. In the field setting, a core temperature should be measured rectally, as this is more accurate than axillary, temporal, oral, and aural temperature measurements. In the hospital setting, an esophageal temperature probe may be placed in intubated patients and provides the most accurate measurement of core body temperature. Acceptable alternatives include a continuous temperature-sensing indwelling urinary catheter or an indwelling rectal temperature probe [49].

It is important to consider diagnostic evaluation for alternative etiologies of hyperthermia and altered mental status. In the prehospital setting, point-of-care glucose testing and point-of-care electrolyte measurement with a handheld blood analyzer should be considered. In the hospital setting, laboratory evaluation to evaluate for sepsis, elevated inflammatory markers, electrolyte derangements, acute hepatic injury, acute renal injury, hyperuricemia, acute lung injury (blood gas measurement), creatinine kinase levels, thyroid derangement, ethanol levels, salicylate levels, coagulopathic and hemolytic evaluation, urinalysis, and urine drug screen should be performed. There should be consideration of blood cultures if infection is suspected as a component [50]. Cardiac evaluation with an electrocardiogram, cardiac biomarkers, and telemetry monitoring to evaluate for an underlying cardiac etiology of an altered level of consciousness should be considered [51]. A chest radiograph can help evaluate for acute lung injury. A complete blood count is non-specific but may show leukocytosis and/or an increased neutrophil-to-lymphocyte ratio, while a metabolic panel may reveal elevated creatinine and hyperkalemia [2,52]. Elevations in liver-associated enzymes portend more serious pathology, and elevations in creatinine kinase suggest development of rhabdomyolysis, also confirmed on urinalysis [53].

What Other Dysfunctions Can Be Seen in Heat Stroke?

The primary dysfunction seen in heatstroke is central nervous system dysfunction [54]. This may include behavioral changes, confusion, irritability, impaired level of consciousness, coma, delirium, ataxia, and convulsions [54]. In the setting of prolonged hyperthermia, heatstroke can progress to multiorgan dysfunction. Cardiovascular dysfunction may include sinus tachycardia and hemodynamic instability. 

Electrocardiographically, tachydysrhythmias, QT prolongation, and ST segment and T wave changes can also be seen [55,56]. Respiratory dysfunction can progress from tachypnea to respiratory failure requiring mechanical ventilation. Hematologic dysfunction includes disseminated intravascular coagulopathy and is associated with poor outcomes [17]. Similarly tied to negative prognostic outcomes, hepatic impairment may develop with elevated liver aminotransferases and can progress to acute liver failure [57].

Renal impairment with acute kidney injury (AKI) is a frequent and prognostically significant complication of heatstroke, warranting further discussion [58]. In a U.S. national analysis of 3,372 heat stroke hospitalizations, renal failure was the most common end-organ failure, with 2% of patients requiring renal replacement therapy and an overall in-hospital mortality of 5%; the presence of end-organ failure was independently associated with increased mortality [52]. The pathophysiology is multifactorial, arising from prerenal hypovolemia and redistribution of blood flow to the skin, reducing renal perfusion [6,7]. Additionally, rhabdomyolysis, the most common complication of heat stroke hospitalization, releases myoglobin that is directly nephrotoxic [14,52]. Neutrophil extracellular trap-mediated immunothrombus formation and disseminated intravascular coagulopathy can further compound microvascular renal injury [14]. Heat-induced gastrointestinal barrier dysfunction with resultant endotoxemia amplifies systemic organ damage [8,13]. Fluids are commonly given for AKIs and heat stroke in the hospital setting. However, a fluid resuscitation strategy may influence AKI risk. In a 2024 retrospective study, Chen and colleagues found the volume of crystalloid (normal saline; NS) administered in the emergency department was associated with increased AKI incidence in heat stroke patients [58]. Specifically, they found the volume of NS infusion was associated with the incidence of AKI (OR, 2.51; 95% CI, 1.43-4.40; p = .001), admission to the ICU (OR, 3.46; 95% CI, 1.58-7.54; p = .002), and length of stay in the ICU (β, 1.00 days; 95% CI, 0.44-1.56; p < .001) and hospital (β, 1.41 days; 95% CI, 0.37-2.45; p = .008). This highlights that while prerenal hypovolemia contributes to renal damage, central venous pressure monitoring has demonstrated that most heatstroke patients are not profoundly fluid-depleted [25], and judicious fluid resuscitation should be observed. Prognosis worsens when kidney dysfunction is sustained, particularly in the late renal-hepatic phase beyond 96 hours [35].

Heat stress and kidney injury represent a growing concern in the context of climate change. The increasing frequency and intensity of heatwaves have substantially elevated the burden of heat-related illness globally [7,20]. Kidney injury from heat exposure is not limited to acute heatstroke; chronic dehydration and recurrent subclinical heat stress can lead to kidney fibrosis and chronic kidney disease, a pattern increasingly recognized among outdoor workers in equatorial regions [7,11]. Organ dysfunction can persist for years following heat-related injury, with affected individuals at two- to three-times greater risk of death for decades after the initial insult [7]. As global temperatures continue to rise, the burden of both acute and chronic heat-related kidney disease is expected to increase, underscoring the importance of early recognition, aggressive cooling, and long-term renal surveillance in heatstroke survivors [7,54].

What Are the Recommended Treatments for Heatstroke in the Prehospital Setting?

Once heatstroke is recognized, treatment of heatstroke should be initiated immediately in the field, and Emergency Medical Services (EMS) should be activated for transportation of the patient to a medical treatment facility. As opposed to the “scoop and run” approach, a “cool and run” approach should be prioritized with a goal of rapidly cooling patients to 39°C [2]. Outdoor laborers who lived in an area without EMS protocols to "cool first, transport second" were found to have 3.7 times higher odds of mortality than those who had these protocols in place [59]. Any life-threatening complications that compromise airway, breathing, and circulation should be addressed, following standard Advanced Cardiac Life Support protocols, and cardiopulmonary resuscitation should be administered if indicated.

Passive cooling begins with removing the patient from exposure to the heat and out of exposure to the sun if possible or pertinent. Direct contact with the hot ground should be avoided with a physical barrier. Additionally, the patient’s clothing should be loosened or removed to allow for heat exchange. 

Active cooling in the field is most effectively achieved with conductive cooling via cold-water immersive therapies, and this is the optimal method of cooling in exertional heatstroke [26]. To accomplish this, the patient’s entire torso and extremities should be submerged in the ice water. Care should be taken to ensure that the patient’s head remains above water and their airway does not become compromised. Of note, while not standard, there is a documented case of a patient undergoing successful and safe electrical cardioversion while submerged in an immersive ice bath [60]. If an immersive ice bath is not logistically possible, the patient can be laid out onto a tarp or plastic sheet, and ice and water can be poured onto the sheet with the sides folded upright and consistently agitated, known as “tarp-assisted cooling” or the “tarp taco” [61]. In the absence of ice or limited supplies, natural bodies of water nearby can be used for cooling; however, the patient must be closely attended to for airway protection and prevention of drowning. Alternatively, a pit can be dug in a shaded area, lined with a tarp or canvas, and filled with water as a makeshift cool-water immersive bath.

Conductive cooling can be achieved via other techniques in the field [62]. A common method for conductive cooling is the application of ice sheets to the patient. Ice sheets are linen sheets or towels that are submerged in freezing water and subsequently applied in direct contact to the patient’s body in the axillae, groin, and neck, and rolled around the patient. To remain effective, these should be changed approximately every 2-4 minutes [63,64]. A study of marathon runners showed that this method of cooling can effectively reduce a person's core temperature to <40°C within 30 minutes [64]. If ice sheets are not available, ice packs should be applied to the patient’s axilla, groin, and neck. Alternatively, the patient can be repeatedly doused with cool water. If fans are available, fanning the patient in combination with ice sheets or ice packs can expedite cooling through evaporative measures. 

Aggressive cooling measures should be continued until mental status normalizes and/or core temperature reaches 39°C, a physiologic temperature in which the body’s natural cooling methods can begin to function normally again.

What Are the Recommended Passive Cooling Techniques for Heatstroke in the Hospital Setting?

Like the aforementioned techniques, passive cooling in the hospital setting focuses on immersive techniques if available, with evaporative techniques as an additional option. Of note, these methods are a temporizing measure for the first few hours to help rapidly correct hyperthermia. Regardless of the modality chosen, the crucial first step is to remove all clothing and avoid covering the patient in sheets or other material, which can impede heat transfer and loss. The ideal modality for in-hospital passive cooling is an immersive bath, which can effectively cool at a rate of 0.15-0.35°C/min due to the total body surface contact of skin and water and has been shown to have superior outcomes compared to modalities of cooling [38]. However, these are usually deeper baths employed for long-duration exertional events (e.g., marathons) and are not likely to be readily available in most EDs. However, some hospitals have implemented a protocol for submersion in the ED setting [2,39]. Due to the nature of critical illness, a prone system will allow for intubation and continued resuscitation, which can be accomplished either by utilizing a body bag, which is readily available in the hospital setting, or with multiple tarps [1,65]. One center implemented a cold water immersion protocol [39] with good success, with 68% (41/60 patients) surviving to hospital discharge [65]. Notably, a significant portion of these patients had concurrent substance use (78.3%), helping to promote cold water immersion as an effective treatment for toxicological elevations in temperature, not just exertional heatstroke [66].

If immersive techniques are not possible, evaporative cooling can provide passive cooling at approximately 0.1°C/hr, which is only half as effective as immersive strategies [2,38]. Once fully undressed, the patient should be sprayed with tepid water using a spray bottle or atomizer to generate small water droplets to maximize evaporative heat loss. While commercially available, adaptive cooling systems are challenging to implement with limited utility as compared to less technologically complex strategies, which may delay the rapid need to cool patients.

These similar evaporative techniques can be employed in the nasotracheal passages for adjunctive assistance in cooling, capitalizing on evaporative cooling of water molecules within the respiratory tract [67]. In patients without concurrent respiratory issues, dry air is run at 20-40 L/min into the nasal passages, avoiding oxygen to prevent hyperoxia and oxygen toxicity, though oxygen may be run within a blender [68]. Additionally, for patients who require positive pressure or are in hypoxemic respiratory failure, continuous positive airway pressure (CPAP) can be utilized, though the heat moisture exchanger (HME) should be removed to facilitate evaporative cooling [68,69]. One limitation of this technique is the duration of utilization, as this evaporative cooling will dry out the airway and nasal passages.

What Are the Recommended Active Cooling Techniques for Heatstroke in the Hospital Setting?

Aggressive cooling measures should continue in the hospital setting until the core temperature reaches 39°C [38]. Conductive cooling via cold water immersion or application of ice packs to the patient should be continued in the hospital setting. There are also numerous manufactured surface cooling devices (SCDs) that may assist with cooling (e.g., Arctic Sun, ZOLL STx™ Surface Pad System, Blanketrol III). Alternatively, a combination of convective and evaporative cooling measures can be utilized. The patient may have tepid water applied to their body with sponges, gauze sheets, or a spray bottle and fans to produce air circulation that enables evaporation of water from the patient’s skin. This is often better tolerated in elderly and agitated patients than immersive cooling.

Invasive cooling measures may be performed in the hospital setting. The easiest form of invasive cooling to implement is the administration of chilled, isotonic intravenous fluids at 4°C, which can provide adjunctive cooling when used in conjunction with other methodologies; however, it is insufficient as a primary method of cooling [70]. Other more aggressive forms of invasive cooling include the use of body cavity lavage with chilled fluids (e.g., gastric lavage, bladder lavage, thoracic lavage via chest tubes, and rectal lavage). These have been utilized but have minimal data to support them as a primary treatment for heatstroke, although they have been used successfully in extreme cases [71]. Intravascular cooling devices have been approved in the United States for targeted temperature management after cardiac arrest or neurosurgery; however, there is less data to support this practice with rapid, efficacious cooling [72], and their use should not distract from less technologically complex methods of rapid cooling.

There are no effective pharmacologic measures for the treatment of heatstroke. Antipyretics, such as aspirin, nonsteroidal anti-inflammatory drugs (NSAIDs), and acetaminophen, are ineffective in the treatment of heatstroke as they mitigate hyperthermia by inhibiting the production of cyclooxygenase as mediated by the hypothalamus, which does not play a role in classic and exertional heatstroke [12,73]. Unless otherwise indicated, antipyretics should be avoided, as the patient with heatstroke is at high risk of both liver and renal damage. While effective at reducing temperature from malignant hyperthermia, there is no evidence to support the use of dantrolene in heatstroke [74].

What Considerations Are There for the Management of Hemodynamic Instability in Heatstroke?

Despite the potentially similar clinical appearance to phenotypes of distributive shock, the management of hemodynamically unstable heatstroke differs significantly. Unlike early norepinephrine use in distributive shock after an appropriate fluid trial, persistent hypotension or hemodynamic instability in heatstroke requires continued administration of cooled fluid and avoidance of vasopressors. The alpha-adrenergic agonism secondary to any exogenous catecholamine, such as norepinephrine, serves to further vasoconstrict cutaneous capillary beds, further hindering heat transfer and cooling [50]. In contrast to vasopressors in heatstroke, administration of cold fluid allows for continued cooling, whereas surface cooling may be less effective in heat transfer for hemodynamically unstable patients due to the delay in heat transfer [43]. Should the patient remain persistently hypotensive despite aggressive, active cooling efforts, vasopressors may be considered for maintaining a mean arterial pressure greater than 65 mm Hg, though this decision should be significantly delayed as compared to current sepsis management [50]. This management difference highlights the importance of recognition and avoiding anchoring to best resuscitate heatstroke patients.

## Conclusions

Heat-related illness exists on a spectrum. Heat stroke represents a true medical emergency, requiring rapid recognition and decisive management. For emergency clinicians, early identification of hyperthermia with central nervous system dysfunction is crucial. Prompt initiation of aggressive cooling remains the most critical determinant of outcome. Many toxicologic, neurologic, endocrine, and infectious conditions can mimic heat stroke. Clinicians must pursue a focused diagnostic evaluation while avoiding delays in definitive treatment. Management should prioritize rapid reduction of core temperature to 39°C using the fastest available modality. Ideally, cold water immersion is preferred. Simultaneously, clinicians should address agitation, airway needs, and evolving multiorgan dysfunction. Supportive care is often needed. This includes fluid resuscitation and close monitoring for complications such as rhabdomyolysis, coagulopathy, hepatic injury, and renal failure. Commonly used antipyretic medications have no role in treatment. They should not distract from rapid physical cooling strategies. Severe heat stroke can cause rapid clinical deterioration. Because of high morbidity and mortality, most patients require admission to an intensive care unit for ongoing monitoring, organ support, and management of complications. A systematic emergency department approach that emphasizes rapid cooling, thoughtful resuscitation, and early recognition of complications can substantially reduce morbidity and mortality from heat stroke.

## References

[1] Lipman GS, Gaudio FG, Eifling KP (2019). Wilderness medical society clinical practice guidelines for the prevention and treatment of heat illness: 2019 update. Wilderness Environ Med.

[2] Kravchenko J, Abernethy AP, Fawzy M, Lyerly HK (2013). Minimization of heatwave morbidity and mortality. Am J Prev Med.

[3] Bouchama A, Dehbi M, Mohamed G (2007). Prognostic factors in heat wave related deaths: a meta-analysis. Arch Intern Med.

[4] Hajat S, O'Connor M, Kosatsky T (2010). Health effects of hot weather: from awareness of risk factors to effective health protection. Lancet.

[5] Backer HD, Shopes E, Collins SL, Barkan H (1999). Exertional heat illness and hyponatremia in hikers. Am J Emerg Med.

[6] Khosla R, Guntupalli KK (1999). Heat-related illnesses. Crit Care Clin.

[7] Ebi KL, Capon A, Berry P (2021). Hot weather and heat extremes: health risks. Lancet.

[8] Savioli G, Zanza C, Longhitano Y (2022). Heat-related illness in emergency and critical care: recommendations for recognition and management with medico-legal considerations. Biomedicines.

[9] Charkoudian N (2016). Human thermoregulation from the autonomic perspective. Auton Neurosci.

[10] Charkoudian N (2003). Skin blood flow in adult human thermoregulation: how it works, when it does not, and why. Mayo Clin Proc.

[11] Chapman CL, Schlader ZJ (2025). Extreme heat stress in older adults: A punch to the gut, kidneys or more?. Exp Physiol.

[12] Baker LB (2019). Physiology of sweat gland function: The roles of sweating and sweat composition in human health. Temperature (Austin).

[13] Cantet JM, Yu Z, Ríus AG (2021). Heat stress-mediated activation of immune-inflammatory pathways. Antibiotics (Basel).

[14] Iba T, Connors JM, Levi M, Levy JH (2022). Heatstroke-induced coagulopathy: Biomarkers, mechanistic insights, and patient management. EClinicalMedicine.

[15] Chalise SN, Mirza E, Malik R (2023). Heat stroke leading to a fatal outcome. Cureus.

[16] Gautret P, Bauge M, Simon F (2013). Overweight and obesity in French Hajj pilgrims. J Immigr Minor Health.

[17] Wu X, Brady JE, Rosenberg H, Li G (2014). Emergency department visits for heat stroke in the United States, 2009 and 2010. Inj Epidemiol.

[18] (2026). Centers of Disease Control and Prevention: Heat-related illnesses and death -- United States, 1994-1995. MMWR Morb Mortal Wkly Rep.

[19] AU MG, BE JW (1956). Observations on one hundred cases of heatstroke. J Am Med Assoc.

[20] The Lancet (2021). Health in a world of extreme heat. Lancet.

[21] Dematte JE, O'Mara K, Buescher J (1998). Near-fatal heat stroke during the 1995 heat wave in Chicago. Ann Intern Med.

[22] Argaud L, Ferry T, Le QH (2007). Short- and long-term outcomes of heatstroke following the 2003 heat wave in Lyon, France. Arch Intern Med.

[23] Misset B, De Jonghe B, Bastuji-Garin S (2006). Mortality of patients with heatstroke admitted to intensive care units during the 2003 heat wave in France: a national multiple-center risk-factor study. Crit Care Med.

[24] Guerra KC, Toncar A, Krishnamurthy K (2025). Miliaria. StatPearls.

[25] Seraj MA, Channa AB, Al Harthi SS (1991). Are heat stroke patients fluid depleted? Importance of monitoring central venous pressure as a simple guideline for fluid therapy. Resuscitation.

[26] Bouchama A, Dehbi M, Chaves-Carballo E (2007). Cooling and hemodynamic management in heatstroke: practical recommendations. Crit Care.

[27] Samarasinghe JL (2001). Heat stroke in young adults. Trop Doct.

[28] Meier K, Lee K (2017). Neurogenic fever. J Intensive Care Med.

[29] Kamidani R, Okada H, Kitagawa Y (2021). Severe heat stroke complicated by multiple cerebral infarctions: a case report. J Med Case Rep.

[30] Wesdock JC, Donoghue AM (2019). Life-threatening heat-related illness with severe hyponatremia in an aluminum smelter worker. Am J Ind Med.

[31] Hausfater P, Mégarbane B, Fabricatore L (2012). Serum sodium abnormalities during nonexertional heatstroke: incidence and prognostic values. Am J Emerg Med.

[32] Hongjun K, Qing S, Yan Z (2015). Fluid resuscitation and standard drug treatment strategies in heatstroke Chinese patients. Drug Res (Stuttg).

[33] Hee-Nee P, Rupeng M, Lee VJ (2010). Treatment of exertional heat injuries with portable body cooling unit in a mass endurance event. Am J Emerg Med.

[34] Kew M, Bersohn I, Seftel H (1971). The diagnostic and prognostic significance of the serum enzyme changes in heatstroke. Trans R Soc Trop Med Hyg.

[35] Pease S, Bouadma L, Kermarrec N (2009). Early organ dysfunction course, cooling time and outcome in classic heatstroke. Intensive Care Med.

[36] Matsumoto H, Takeba J, Umakoshi K (2019). Successful treatment for disseminated intravascular coagulation (DIC) corresponding to phenotype changes in a heat stroke patient. J Intensive Care.

[37] Farooqi S, Raj S, Koyfman A, Long B (2023). High risk and low prevalence diseases: thyroid storm. Am J Emerg Med.

[38] Barletta JF, Palmieri TL, Toomey SA (2025). Society of critical care medicine guidelines for the treatment of heat stroke. Crit Care Med.

[39] Comp G, Pugsley P, Sklar D (2025). Heat stroke management updates: a description of the development of a novel in-emergency department cold-water immersion protocol and guide for implementation. Ann Emerg Med.

[40] Poulton T, Walker R (1987). Helicopter cooling of heatstroke victims. Aviat Space Environ Med.

[41] Adams WM, Butke EE, Lee J, Zaplatosch ME (2021). Cooling capacity of transpulmonary cooling and cold-water immersion after exercise-induced hyperthermia. J Athl Train.

[42] Kim F, Nichol G, Maynard C (2014). Effect of prehospital induction of mild hypothermia on survival and neurological status among adults with cardiac arrest: a randomized clinical trial. JAMA.

[43] Rajek A, Greif R, Sessler DI (2000). Core cooling by central venous infusion of ice-cold (4 degrees C and 20 degrees C) fluid: isolation of core and peripheral thermal compartments. Anesthesiology.

[44] Hamaya H, Hifumi T, Kawakita K (2015). Successful management of heat stroke associated with multiple-organ dysfunction by active intravascular cooling. Am J Emerg Med.

[45] Thangavelu R, George SK, Kandasamy R (2020). Prophylactic low dose ketamine infusion for prevention of shivering during spinal anesthesia: A randomized double blind clinical trial. J Anaesthesiol Clin Pharmacol.

[46] Jain A, Gray M, Slisz S (2018). Shivering treatments for targeted temperature management: a review. J Neurosci Nurs.

[47] Otta JJ, Ohden L, Bell DG (2021). Malignant hyperthermia-associated rhabdomyolysis after succinylcholine: a case report. S D Med.

[48] Yuan R, Wang L, Deng ZH (2023). Protective effects of mesenchymal stem cells against central nervous system injury in heat stroke. Curr Stem Cell Res Ther.

[49] Umińska JM, Buszko K, Ratajczak J (2020). Comparison of temperature measurements in esophagus and urinary bladder in comatose patients after cardiac arrest undergoing mild therapeutic hypothermia. Cardiol J.

[50] Marchand M, Gin K (2022). The cardiovascular system in heat stroke. CJC Open.

[51] Bao CH, Feng Q, Zhang C (2024). Heat stroke with significantly elevated troponin and dynamic ECG changes: Myocardial infarction or myocardial injury?. Am J Med Sci.

[52] Thongprayoon C, Qureshi F, Petnak T (2020). Impact of acute kidney injury on outcomes of hospitalizations for heat stroke in the United States. Diseases.

[53] Ribeiro F, Bibi M, Pereira M (2020). Severe acute liver injury related to heat stroke. Eur J Case Rep Intern Med.

[54] Patel J, Boyer N, Mensah K (2023). Critical illness aspects of heatstroke: a hot topic. J Intensive Care Soc.

[55] Case D, Harrigan R (2015). Heat stroke-induced sinoatrial node dysfunction. J Emerg Med.

[56] Doshi HH, Giudici MC (2015). The EKG in hypothermia and hyperthermia. J Electrocardiol.

[57] Wagner M, Kaufmann P, Fickert P (2003). Successful conservative management of acute hepatic failure following exertional heatstroke. Eur J Gastroenterol Hepatol.

[58] Chen L, Zhao J, Lu L (2024). Association between normal saline infusion volume in the emergency department and acute kidney injury in heat stroke patients: a multicenter retrospective study. Ren Fail.

[59] Tishukaj F, Stearns RL, Morrissey MC (2024). Exertional heat stroke best practices in U.S. emergency medical services guidelines. J Emerg Med.

[60] Feinstein B, Kelley J, Blackburn P, Connell P (2023). Synchronized cardioversion performed during cold water immersion of a heatstroke patient. Ann Emerg Med.

[61] Tucker L, Evans E (2023). Heatstroke on the rise: a guide to implementing tarp-assisted cooling with oscillation (TACO) in the emergency department. Adv Emerg Nurs J.

[62] Gaudio FG, Grissom CK (2016). Cooling methods in heat stroke. J Emerg Med.

[63] DeGroot DW, Henderson KN, O'Connor FG (2023). Cooling modality effectiveness and mortality associate with prehospital care of exertional heat stroke casualities. J Emerg Med.

[64] Dollée N, Alsma J, Goedhart R (2025). Exertional heat stroke: are we cool enough? Retrospective observational study of patients of running events. J Emerg Med.

[65] Kim DA, Lindquist BD, Shen SH (2020). A body bag can save your life: a novel method of cold water immersion for heat stroke treatment. J Am Coll Emerg Physicians Open.

[66] Stowell JR, Pugsley P, McElhinny M (2025). Emergency department management of acute heatstroke: a retrospective analysis from Phoenix, Arizona. West J Emerg Med.

[67] Chava R, Zviman M, Raghavan MS (2017). Rapid induction of therapeutic hypothermia using transnasal high flow dry air. Ther Hypothermia Temp Manag.

[68] Chava R, Zviman M, Assis FR (2019). Effect of high flow transnasal dry air on core body temperature in intubated human subjects. Resuscitation.

[69] Ziai WC, Shah D, Assis FR (2019). Feasibility and safety of transnasal high flow air to reduce core body temperature in febrile neurocritical care patients: a pilot study. Neurocrit Care.

[70] Chen L, Liu C, Zhang Z (2025). Efficacy of cooling blankets and cold fluid infusions in classic heatstroke treatment: a controlled study in rats. J Inflamm Res.

[71] Smith JE (2005). Cooling methods used in the treatment of exertional heat illness. Br J Sports Med.

[72] Yokobori S, Koido Y, Shishido H (2018). Feasibility and safety of intravascular temperature management for severe heat stroke: a prospective multicenter pilot study. Crit Care Med.

[73] Fujii N, Pastore OL, McGarr GW (2018). Cyclooxygenase-1 and -2 modulate sweating but not cutaneous vasodilation during exercise in the heat in young men. Physiol Rep.

[74] Hausfater P (2005). Dantrolene and heatstroke: a good molecule applied in an unsuitable situation. Crit Care.

